# Supplementary material for: Modeling glioblastoma heterogeneity as a dynamic network of cell states
Source: Mol Syst Biol. 2021 Sep 16;17(9):e10105. doi: 10.15252/msb.202010105 (PMC8444284; doi:10.15252/msb.202010105)
Supplement: Supplementary file 5 — Source Data for Figure 3 [file MSB-17-e10105-s001.zip › Figure3A_sourcedata/GSEA_3065/hallmarks_state5.GseaPreranked.1623416302988/HALLMARK_HYPOXIA.html]

Details for gene set HALLMARK\_HYPOXIA[GSEA]

|  || Dataset | state5 |
| Phenotype | NoPhenotypeAvailable |
| Upregulated in class | na\_neg |
| GeneSet | HALLMARK\_HYPOXIA |
| Enrichment Score (ES) | -0.29114482 |
| Normalized Enrichment Score (NES) | -1.0430341 |
| Nominal p-value | 0.375 |
| FDR q-value | 0.68545026 |
| FWER p-Value | 1.0 |
Table: GSEA Results Summary

  

Fig 1: Enrichment plot: HALLMARK\_HYPOXIA      
 Profile of the Running ES Score & Positions of GeneSet Members on the Rank Ordered List

  

| PROBE | GENE SYMBOL | GENE\_TITLE | RANK IN GENE LIST | RANK METRIC SCORE | RUNNING ES | CORE ENRICHMENT || 1 | WSB1 |  |  | 13 | 0.861 | 0.0342 | No |
| 2 | BCAN |  |  | 15 | 0.850 | 0.0692 | No |
| 3 | HSPA5 |  |  | 43 | 0.698 | 0.0951 | No |
| 4 | GPC1 |  |  | 98 | 0.530 | 0.1109 | No |
| 5 | HEXA |  |  | 369 | 0.352 | 0.0950 | No |
| 6 | CXCR4 |  |  | 450 | 0.325 | 0.0995 | No |
| 7 | STC1 |  |  | 530 | 0.303 | 0.1031 | No |
| 8 | PRKCA |  |  | 652 | 0.275 | 0.1008 | No |
| 9 | ZNF292 |  |  | 656 | 0.274 | 0.1118 | No |
| 10 | SDC3 |  |  | 728 | 0.255 | 0.1143 | No |
| 11 | ILVBL |  |  | 757 | 0.249 | 0.1215 | No |
| 12 | P4HA1 |  |  | 792 | 0.242 | 0.1277 | No |
| 13 | HS3ST1 |  |  | 814 | 0.238 | 0.1352 | No |
| 14 | PAM |  |  | 844 | 0.233 | 0.1415 | No |
| 15 | ANKZF1 |  |  | 879 | 0.228 | 0.1472 | No |
| 16 | TGFBI |  |  | 1177 | 0.188 | 0.1214 | No |
| 17 | GAA |  |  | 1182 | 0.187 | 0.1287 | No |
| 18 | SLC2A1 |  |  | 1190 | 0.186 | 0.1356 | No |
| 19 | SDC4 |  |  | 1193 | 0.186 | 0.1431 | No |
| 20 | SLC2A3 |  |  | 1209 | 0.183 | 0.1490 | No |
| 21 | SERPINE1 |  |  | 1221 | 0.182 | 0.1553 | No |
| 22 | GPC4 |  |  | 1288 | 0.173 | 0.1550 | No |
| 23 | COL5A1 |  |  | 1328 | 0.168 | 0.1576 | No |
| 24 | SRPX |  |  | 1409 | 0.161 | 0.1552 | No |
| 25 | FOS |  |  | 1412 | 0.161 | 0.1616 | No |
| 26 | EGFR |  |  | 1547 | 0.147 | 0.1526 | No |
| 27 | VEGFA |  |  | 1634 | 0.139 | 0.1486 | No |
| 28 | ERO1A |  |  | 1804 | 0.127 | 0.1348 | No |
| 29 | KDELR3 |  |  | 1926 | 0.117 | 0.1259 | No |
| 30 | P4HA2 |  |  | 2022 | 0.110 | 0.1198 | No |
| 31 | IGFBP3 |  |  | 2037 | 0.109 | 0.1227 | No |
| 32 | NDST1 |  |  | 2132 | 0.103 | 0.1164 | No |
| 33 | CHST3 |  |  | 2253 | 0.094 | 0.1067 | No |
| 34 | TMEM45A |  |  | 2322 | 0.091 | 0.1028 | No |
| 35 | VHL |  |  | 2369 | 0.088 | 0.1013 | No |
| 36 | AKAP12 |  |  | 2478 | 0.081 | 0.0924 | No |
| 37 | SLC37A4 |  |  | 2484 | 0.081 | 0.0952 | No |
| 38 | ATP7A |  |  | 2505 | 0.079 | 0.0962 | No |
| 39 | ADORA2B |  |  | 2563 | 0.076 | 0.0929 | No |
| 40 | SLC6A6 |  |  | 2565 | 0.076 | 0.0959 | No |
| 41 | SCARB1 |  |  | 2808 | 0.062 | 0.0712 | No |
| 42 | PLAUR |  |  | 2826 | 0.061 | 0.0718 | No |
| 43 | B3GALT6 |  |  | 2870 | 0.059 | 0.0694 | No |
| 44 | CA12 |  |  | 2885 | 0.058 | 0.0702 | No |
| 45 | NAGK |  |  | 2896 | 0.058 | 0.0715 | No |
| 46 | DTNA |  |  | 3219 | 0.040 | 0.0368 | No |
| 47 | CCNG2 |  |  | 3262 | 0.038 | 0.0336 | No |
| 48 | TPST2 |  |  | 3303 | 0.036 | 0.0306 | No |
| 49 | STC2 |  |  | 3306 | 0.036 | 0.0319 | No |
| 50 | KLF6 |  |  | 3447 | 0.029 | 0.0173 | No |
| 51 | KLHL24 |  |  | 3502 | 0.027 | 0.0123 | No |
| 52 | MXI1 |  |  | 3791 | 0.016 | -0.0195 | No |
| 53 | VLDLR |  |  | 3854 | 0.014 | -0.0259 | No |
| 54 | IDS |  |  | 4033 | 0.006 | -0.0458 | No |
| 55 | TPBG |  |  | 4075 | 0.004 | -0.0502 | No |
| 56 | ATF3 |  |  | 4225 | -0.002 | -0.0670 | No |
| 57 | MAP3K1 |  |  | 4259 | -0.004 | -0.0705 | No |
| 58 | KDM3A |  |  | 4280 | -0.005 | -0.0726 | No |
| 59 | GBE1 |  |  | 4323 | -0.007 | -0.0770 | No |
| 60 | SDC2 |  |  | 4596 | -0.018 | -0.1070 | No |
| 61 | PGM2 |  |  | 4885 | -0.029 | -0.1383 | No |
| 62 | UGP2 |  |  | 5013 | -0.034 | -0.1513 | No |
| 63 | SIAH2 |  |  | 5063 | -0.035 | -0.1553 | No |
| 64 | BTG1 |  |  | 5087 | -0.037 | -0.1564 | No |
| 65 | PPP1R15A |  |  | 5321 | -0.045 | -0.1808 | No |
| 66 | DDIT3 |  |  | 5335 | -0.046 | -0.1804 | No |
| 67 | ERRFI1 |  |  | 5451 | -0.050 | -0.1913 | No |
| 68 | RBPJ |  |  | 5474 | -0.051 | -0.1917 | No |
| 69 | JMJD6 |  |  | 5502 | -0.053 | -0.1925 | No |
| 70 | NDRG1 |  |  | 5645 | -0.060 | -0.2061 | No |
| 71 | XPNPEP1 |  |  | 5652 | -0.060 | -0.2043 | No |
| 72 | ISG20 |  |  | 5757 | -0.064 | -0.2134 | No |
| 73 | FOSL2 |  |  | 5785 | -0.065 | -0.2137 | No |
| 74 | CDKN1C |  |  | 5823 | -0.066 | -0.2151 | No |
| 75 | ENO2 |  |  | 5895 | -0.069 | -0.2203 | No |
| 76 | ALDOA |  |  | 5909 | -0.070 | -0.2189 | No |
| 77 | BHLHE40 |  |  | 5950 | -0.072 | -0.2204 | No |
| 78 | CDKN1B |  |  | 5996 | -0.074 | -0.2224 | No |
| 79 | DUSP1 |  |  | 5997 | -0.074 | -0.2194 | No |
| 80 | IRS2 |  |  | 6033 | -0.076 | -0.2202 | No |
| 81 | NR3C1 |  |  | 6073 | -0.078 | -0.2213 | No |
| 82 | PLIN2 |  |  | 6129 | -0.081 | -0.2242 | No |
| 83 | FOXO3 |  |  | 6238 | -0.087 | -0.2328 | No |
| 84 | HK1 |  |  | 6420 | -0.097 | -0.2492 | No |
| 85 | DPYSL4 |  |  | 6520 | -0.102 | -0.2561 | No |
| 86 | CHST2 |  |  | 6560 | -0.106 | -0.2562 | No |
| 87 | HMOX1 |  |  | 6561 | -0.106 | -0.2518 | No |
| 88 | TIPARP |  |  | 6731 | -0.116 | -0.2661 | No |
| 89 | GLRX |  |  | 6822 | -0.123 | -0.2712 | No |
| 90 | PFKP |  |  | 6845 | -0.124 | -0.2685 | No |
| 91 | NEDD4L |  |  | 6876 | -0.128 | -0.2666 | No |
| 92 | DDIT4 |  |  | 6968 | -0.135 | -0.2713 | No |
| 93 | CITED2 |  |  | 7120 | -0.147 | -0.2823 | No |
| 94 | PFKL |  |  | 7197 | -0.155 | -0.2844 | Yes |
| 95 | CASP6 |  |  | 7206 | -0.156 | -0.2789 | Yes |
| 96 | MYH9 |  |  | 7248 | -0.161 | -0.2768 | Yes |
| 97 | PGM1 |  |  | 7268 | -0.164 | -0.2722 | Yes |
| 98 | TPD52 |  |  | 7309 | -0.168 | -0.2698 | Yes |
| 99 | RRAGD |  |  | 7321 | -0.170 | -0.2640 | Yes |
| 100 | HOXB9 |  |  | 7336 | -0.172 | -0.2584 | Yes |
| 101 | CAV1 |  |  | 7575 | -0.200 | -0.2770 | Yes |
| 102 | SLC25A1 |  |  | 7589 | -0.203 | -0.2701 | Yes |
| 103 | GALK1 |  |  | 7610 | -0.206 | -0.2638 | Yes |
| 104 | CAVIN3 |  |  | 7853 | -0.244 | -0.2811 | Yes |
| 105 | PNRC1 |  |  | 7867 | -0.247 | -0.2723 | Yes |
| 106 | GRHPR |  |  | 7895 | -0.255 | -0.2648 | Yes |
| 107 | JUN |  |  | 7930 | -0.262 | -0.2578 | Yes |
| 108 | SAP30 |  |  | 7936 | -0.263 | -0.2475 | Yes |
| 109 | FAM162A |  |  | 7983 | -0.272 | -0.2414 | Yes |
| 110 | GPI |  |  | 8020 | -0.280 | -0.2339 | Yes |
| 111 | LDHA |  |  | 8263 | -0.343 | -0.2470 | Yes |
| 112 | BNIP3L |  |  | 8266 | -0.344 | -0.2330 | Yes |
| 113 | MIF |  |  | 8296 | -0.353 | -0.2217 | Yes |
| 114 | PGK1 |  |  | 8452 | -0.408 | -0.2223 | Yes |
| 115 | CAVIN1 |  |  | 8657 | -0.531 | -0.2233 | Yes |
| 116 | CDKN1A |  |  | 8679 | -0.545 | -0.2031 | Yes |
| 117 | HDLBP |  |  | 8699 | -0.560 | -0.1821 | Yes |
| 118 | ENO1 |  |  | 8734 | -0.593 | -0.1614 | Yes |
| 119 | MT2A |  |  | 8740 | -0.598 | -0.1372 | Yes |
| 120 | PRDX5 |  |  | 8836 | -0.693 | -0.1193 | Yes |
| 121 | ANXA2 |  |  | 8868 | -0.729 | -0.0926 | Yes |
| 122 | GAPDH |  |  | 8915 | -0.803 | -0.0646 | Yes |
| 123 | TPI1 |  |  | 8939 | -0.852 | -0.0319 | Yes |
| 124 | CSRP2 |  |  | 8954 | -0.891 | 0.0034 | Yes |
Table: GSEA details [plain text format]

  

Fig 2: HALLMARK\_HYPOXIA: Random ES distribution      
 Gene set null distribution of ES for **HALLMARK\_HYPOXIA**
